# The importance of (shared) human values for containing the COVID‐19 pandemic

**DOI:** 10.1111/bjso.12401

**Published:** 2020-06-23

**Authors:** Lukas J. Wolf, Geoffrey Haddock, Antony S. R. Manstead, Gregory R. Maio

**Affiliations:** ^1^ Department of Psychology University of Bath Claverton Down UK; ^2^ School of Psychology Cardiff University UK

**Keywords:** COVID‐19, Corona virus, Human values, Value similarity, Compliance, Prosocial behaviour

## Abstract

The COVID‐19 pandemic poses an exceptional challenge for humanity. Because public behaviour is key to curbing the pandemic at an early stage, it is important for social psychological researchers to use their knowledge to promote behaviours that help manage the crisis. Here, we identify human values as particularly important in driving both behavioural compliance to government guidelines and promoting prosocial behaviours to alleviate the strains arising from a prolonged pandemic. Existing evidence demonstrates the importance of human values, and the extent to which they are shared by fellow citizens, for tackling the COVID‐19 crisis. Individuals who attach higher importance to self‐transcendence (e.g., responsibility) and conservation (e.g., security) values are likely to be more compliant with COVID‐19 behavioural guidelines and to help others who are struggling with the crisis. Further, believing that fellow citizens share one's values has been found to elicit a sense of connectedness that may be crucial in promoting collective efforts to contain the pandemic. The abstract nature of values, and cross‐cultural agreement on their importance, suggests that they are ideally suited to developing and tailoring effective, global interventions to combat this pandemic.

The COVID‐19 pandemic presents humanity with an extraordinary challenge. The rapid spread of the virus and the necessity of waiting for effective treatments or vaccines (BBC, 2020a) highlights the importance of changing human behaviour to contain the pandemic. Accordingly, governments across the world have introduced measures that severely impact individuals' personal and social lives, including closing institutions (e.g., schools, restaurants) and urging people to stay at home, stay away from public places and social gatherings, and work remotely where feasible (e.g., UK Government, 2020). Individuals are also strongly advised to wash their hands regularly and thoroughly, avoid touching objects that others may have touched (e.g., elevator buttons), and keep a 2m distance from others. Data from the United Kingdom suggest that most but not all citizens have complied with these guidelines. As of 3 April, 80% of UK citizens reported avoiding public places, 77% indicated that they improved their personal hygiene (e.g., washing hands), and 57% said they avoided touching objects in public spaces (YouGov, 2020). However, these data also show that a substantial minority does not follow governmental advice, a notion echoed in media reports (e.g., BBC News, 2020b). This non‐compliance by some individuals runs the risk of increasing the likelihood that the virus will continue to spread.

Given that the behaviour of the general public is key to curbing the pandemic at this stage, it is important for social psychological researchers to use their knowledge to promote behaviours that help manage the crisis. Here, we argue that two types of behaviours are of particular significance. First, it is crucial to understand and increase compliance with the guidelines. Many individuals may comply out of a concern for their personal health, but compliance often requires individuals to make sacrifices for the sake of the greater societal good (e.g., engage in self‐isolation in order to protect others). The barrier to engaging in such self‐sacrificial behaviour is increased further by evidence that individuals underestimate the likelihood that they personally will be infected with COVID‐19 (Kuper‐Smith *et al*., 2020). Furthermore, the purpose of the advised self‐sacrificial behaviour, such as protecting the vulnerable and reducing demand on health services, is quite abstract in nature, and the COVID‐19 crisis is likely to require compliance that is sustained over a long period of time. These factors represent additional barriers to achieving the goal of ensuring high levels of sustained compliance.

Second, it is important to help others who are struggling to cope with the crisis. Such prosocial behaviours include doing voluntary work for health services, grocery shopping for vulnerable people, donating to food shelters, and offering support to those who feel overwhelmed. The COVID‐19 pandemic threatens people's sense of well‐being in multiple ways. In addition to the physical threats of possible infection, postponement of non‐urgent (but important) medical treatment, and lack of access to food and other essentials, there are psychological threats posed by (potential) loss of employment, loneliness stemming from isolation, worries about the health of loved ones, and coping with bereavement. Moreover, people who are already struggling with harsh living conditions (e.g., individuals in refugee camps) may be among the worst hit. It is therefore crucial for social psychological research to identify ways to better understand and promote prosocial behaviours to alleviate the strains arising from a prolonged pandemic.

We believe that human values represent a psychological construct that is particularly important in driving both behavioural compliance and prosocial behaviour. This commentary focuses on how behaviour may be influenced by the direct effects of personal values and by perceptions of value similarity or dissimilarity in society.

## Personal values

**Figure 1:**
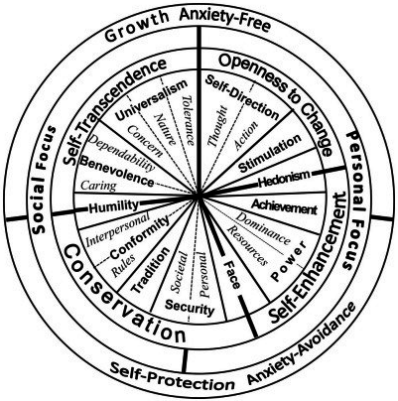
Schwartz's quasi‐circumplex model of values (Schwartz, 1992)

Values are typically defined as abstract goals or guiding principles in people's lives (Maio, 2016; Schwartz, 1992) and have been shown to predict outcomes such as prejudice (Wolf *et al*., 2019), environmental behaviour (Hurst *et al*., 2013), and protest action (Mayton & Furnham, 1994). We focus on the predominant model of values in psychology: The quasi‐circumplex model proposed by Schwartz (1992; Schwartz & Bilsky, 1987, 1990). Its two‐dimensional space contrasts *self‐transcendence* with *self‐enhancement* values (e.g., helpfulness vs achievement) and *openness* with *conservation* values (e.g., freedom vs security; see Figure 1). Self‐transcendence and conservation values are considered as having a social focus, whereas self‐enhancement and openness values are considered as having a personal focus (Schwartz *et al*., 2012). Moreover, self‐transcendence and openness values are conceptualized as anxiety‐free and growth‐oriented, whereas self‐enhancement and conservation values are anxiety‐avoidant and focus on self‐protection. The model's two‐dimensional structure has been replicated in many cross‐sectional and experimental studies conducted in over 80 countries (Bilsky *et al*., 2011; Schwartz *et al*., 2012).

Given that values transcend specific situations, have a structure that is cross‐culturally consistent, and relate to a range of behaviours, there is good reason to believe that they play a widespread role in shaping responses to the COVID‐19 pandemic. Based on the motives that underlie the values in Schwartz's model, one might expect that individuals who endorse values with a social focus, self‐transcendence (e.g., responsibility) and conservation (e.g., family security) values, will show greater compliance with COVID‐19 guidelines, whereas individuals who endorse values with a personal focus, openness (e.g., freedom) and self‐enhancement (e.g., ambition) values, will show lower compliance. People with higher self‐transcendence values should be more likely to comply out of a concern for the safety of others, consistent with the anxiety‐free and other‐oriented focus of such values, whereas those with higher conservation values should be more likely to comply out of a concern for their own safety, consistent with the focus of these values on anxiety‐avoidance and self‐protection. In contrast, it can be expected that individuals who attach higher importance to openness values are likely to struggle with guidelines that restrict their freedom and deprive them of stimulating experiences (e.g., mountain climbing), whereas those who attach higher importance to self‐enhancement values are less likely to adhere to the guidelines when they conflict with their drive for power and achievement.

Consistent with these predictions, there is evidence linking higher conservation values and lower openness values to compliant and security‐oriented behaviour (e.g., Bardi & Schwartz, 2003; Fischer & Smith, 2006; Schwartz *et al*., 2017). However, the social aspect of compliance (i.e., protecting others from the disease) may be relatively unique to pandemic situations, and hence, evidence linking self‐transcendence and self‐enhancement values to behavioural compliance in this context is scarce. Nevertheless, research on COVID‐19 has provided indirect evidence for such a link: Pfattheicher *et al*. (2020) found that empathy relates to a greater motivation to comply with guidelines regarding physical distancing. Across three studies using samples from the United Kingdom, the United States, and Germany, higher empathy predicted and even elicited a stronger motivation to engage in physical distancing. Previous work has linked higher self‐transcendence and lower self‐enhancement values to higher levels of empathy (e.g., Myyrya *et al*., 2010; Silfver *et al*., 2008). Thus, there is initial evidence, albeit indirect, relating increased levels of compliance with COVID‐19 guidelines to higher self‐transcendence and conservation values, and lower self‐enhancement and openness values.

Turning to prosocial behaviour in the context of COVID‐19, such behaviour can also be expected to occur more frequently in individuals higher in self‐transcendence values and lower in self‐enhancement values. This prediction is based on the underlying motives of these values, as discussed above, and on evidence linking these values to a range of prosocial behaviours. For instance, individuals higher in self‐transcendence values are more likely to volunteer to help others, donate money to a prosocial cause, and cooperate rather than compete with others (see Sagiv *et al*., 2017). Such behaviours generally involve putting others' interests first, at some personal cost. There is also evidence that values predict reactions to a disaster (Frink *et al*., 2004). In the context of the 1995 Oklahoma City bombing, individuals higher in self‐transcendence values, and interestingly those higher in conservation values, reported strong increases in macrolevel worries (i.e., concerns about the wider society) immediately after versus before the event. Microlevel worries (i.e., concerns about oneself or close others) also increased over this period among those higher in self‐transcendence and conservation values but only weakly. Given this evidence on increased macrolevel worries, individuals higher in self‐transcendence and conservation values may be more willing to support others struggling with the pandemic, and perhaps not only those others in their immediate community or society, but also those in areas that are particularly badly affected by the crisis (e.g., refugee camps, war zones).

Overall, existing theoretical and empirical work indicates that values play important roles in shaping the likelihood that individuals comply with behavioural requests and engage in prosocial behaviour, both of which are crucial in tackling the COVID‐19 pandemic. This provides insights into the motivations underlying people's compliant and prosocial behaviour and highlights who is particularly likely to be responsive to calls for such behaviours. Building on this, research is urgently needed to identify ways of motivating individuals with higher openness or self‐enhancement values to comply with governmental guidelines in order to enhance protection for vulnerable others. Interventions aimed at changing people's values are likely to be successful but might be short‐lived (e.g., Bernard *et al*., 2003; Blankenship *et al*., 2012), given the temporal stability of values (Bilsky *et al*., 2011). However, it seems plausible that messages tailored to the motives underlying openness and self‐enhancement values will be vital in achieving compliance in these individuals. Research on attitude change has demonstrated that persuasive messages matching aspects of the recipient's attitude (e.g., the attitude's content or function) are more likely to elicit attitude change than mismatched messages (see Haddock & Maio, 2019). Applied to the current context, arguments for COVID‐19 mitigating behaviours may be more effective among individuals higher in openness and self‐enhancement values when the arguments address relevant motives such as stimulation or achievement. For instance, these individuals may be especially responsive to policy recommendations arising from a recent study of public health messages in Italy (Barari *et al*., 2020):

‘We need interventions that make staying following public health protocols more desirable, such as virtual social interactions, online social reading activities, classes, exercise routines, etc. — all designed to reduce the boredom of long term social isolation and to increase the attractiveness of following public health recommendations. Interventions like these will grow in importance as the crisis wears on around the world and staying inside wears on people.' (p.1)

Such messaging appeals to personal growth and stimulation and also reduces anxiety – motives underpinning self‐enhancement and openness values.

At the same time, it may be useful to evaluate the effectiveness of interventions activating self‐transcendence and conservation values (see Sagiv *et al*., 2017). Notwithstanding individual differences, global data robustly indicate that self‐transcendence values and, to a lesser extent, conservation values are particularly important to most people (Schwartz & Bardi, 2001). Consequently, even among those who attach very high importance to self‐enhancement and openness values, it may be beneficial to activate self‐transcendence and conservation values in attempts to motivate behaviour in support of mitigating the pandemic. Although the direct effects of any single‐instance activation are likely to be short‐lived (Maio *et al*., 2009), repeated activation over time may nevertheless break habitual ways of responding. In the United Kingdom, events like the weekly ‘clap for carers’ serve as widespread, repeated social reminders of these values. Such events may serve in the long‐term to highlight how individuals' own principles relate to behaviours such as social distancing or a personal hygiene, thereby potentially helping to sustain public engagement in curbing the spread of the virus. Even for those who do not take part, such events serve as reminders of these values, while helping to correct misperceptions of personal–societal value gaps, which is the second topic in our commentary.

## Value similarities

Values may also be relevant to the COVID‐19 pandemic through the extent to which they are (perceived to be) shared by others. As noted above, a portion of the public is not fully complying with governmental guidelines designed to curb the spread of the virus, and this divide between compliers and non‐compliers is frequently highlighted in media reports (BBC News, 2020b). Such reports may prompt (mis)perceptions of the extent to which one's own value priorities differ from those of fellow citizens. For instance, seeing others ignoring guidelines by gathering in a public place may lead individuals to assume that others assign lower importance to self‐transcendence (e.g., responsibility) or conservation values (e.g., security) than they themselves do. Perceiving others as having different values may reduce people's willingness to self‐sacrifice for the greater societal good because tackling the crisis requires a collective effort. Conversely, perceiving others as sharing one's values may validate them and facilitate collective behaviour, thereby encouraging greater compliance with the guidelines and more prosocial behaviour.

There is recent evidence supporting this view. People's perceptions that others share their values predict and shape a range of outcomes, including a higher sense of connectedness with society (Bernard *et al*., 2006), higher civic engagement (Sanderson *et al*., 2019), and more positive attitudes towards opposing political groups (Hanel & Wolf, 2019) and immigrant groups (e.g., Hanel *et al*., 2019; Wolf *et al*., 2019). These findings build on prior evidence that the adoption of a ‘similarity’ mindset (versus a ‘difference’ mindset) has far‐reaching repercussions for the way one thinks about others (Boer *et al*., 2011; Mussweiler, 2003). Perceived value similarities are therefore an important factor shaping people's sense of connectedness with others, even across group boundaries. Feelings of connectedness, in turn, are likely to increase willingness to do one's share and to assist others, consistent with evidence that feelings of connectedness are linked with higher levels of cooperation in groups (e.g., De Cremer, 2002; De Cremer & Leonardelli, 2003). Together, these findings suggest that socially shared values may be a key binding factor that promotes the collective prosocial behaviour needed in times of crisis.

It is worth noting that the high level of abstraction in human values may be pivotal in this context. Their abstract nature enables individuals to use values as markers of widely shared principles, despite stark differences in cultural and economic background, ideology, and more specific attitudes or behaviours (cf. Boer *et al*., 2011). For instance, festivals of arts, music, science, and sport often attract large audiences in a way that avoids polarizing opinions, while implying solidarity with commonly held values (e.g., creativity, achievement, helpfulness). Accordingly, a focus on widely shared values within and across societies may similarly help tackle a global crisis like COVID‐19. Calls for responsibility and social justice for the vulnerable (i.e., self‐transcendence values), national security (i.e., conservation values), research on COVID‐19 (i.e., openness value), and even introducing war‐time analogies or achievement challenges (i.e., self‐enhancement values) can serve this function of uniting diverse people around common values.

The idea of highlighting value similarities is particularly promising because it is based on evidence: Most people actually *do* possess similar values, in the United Kingdom and beyond (e.g., Hanel *et al*., 2019; Sanderson *et al*., 2019). For instance, despite the strong polarization in the United Kingdom before and after the Brexit referendum, most ‘Leave’ voters and ‘Remain’ voters exhibit substantial overlap in their values (Hanel & Wolf, 2019). However, people often fail to recognize these similarities (Hanel *et al*., 2018; Sanderson *et al*., 2019). To illustrate the point, such recognition failure is evident in the case of environmental values, which most people wrongly assume others endorse less strongly than they do. This bias has been cited as a barrier to environmental change (Bouman & Steg, 2019). Such misperceptions of others' values often stem from a biased inference process that is influenced by salient reports or sights of others' attitudes and behaviours and our own motivated confirmation of existing attitudes (e.g., Hart *et al*., 2009; Kunda, 1990). In this way, we may infer from seeing small groups of individuals (out of hundreds of park visitors) socializing in a park during lockdown that our fellow citizens attach lower importance to self‐transcendence and conservation values than we do.

Given the evidence that we tend to perceive value dissimilarities to be higher than they actually are, it would be beneficial to examine whether correcting such misperceptions would promote more collective behaviour to help mitigate the current pandemic. While previous research has provided factual information on value similarities between groups to correct misperceptions (e.g., Leave voters vs Remain voters; Hanel & Wolf, 2019), we are unaware of research that has highlighted similarities between individuals and societies. Yet, it would be possible to implement this type of intervention on a large scale via interactive integration of values data with feedback on values as reported in national surveys, and this could be deployed rapidly in any of more than 80 nations where such data exist. Given the global nature of the pandemic, this potential for international scale is valuable.

Another interesting issue is whether the effects of such similarities on behaviour apply to all values or only to certain values that are more obviously linked in people's minds to COVID‐19. On the one hand, there is reason to believe that similarity effects should occur across all values, because perceptions of similarity should elicit feelings of common purpose (Boer *et al*., 2011), social connectedness, and validation (Bernard *et al*., 2006; Sanderson *et al*., 2019), regardless of the motive underlying the value. For instance, individuals who perceive others as sharing their values may be more willing to comply because they feel more connected and more motivated to work towards the collective goal of containing the pandemic. On the other hand, individuals may not spontaneously perceive values such as power or achievement to be particularly relevant to the COVID‐19 context. Hence, although perceived similarities in such values may elicit a feeling of validation and connectedness, this feeling may help tackle the pandemic only if the feeling of interconnection *per se*, and not also its underlying values basis, is the primary determinant of collective behaviour mitigating the pandemic. Research would benefit from examining this potential moderation of the impact of value similarity by value type, as well as testing the putative mechanisms of individuals' increased sense of common purpose, validation, and connectedness.

Finally, value similarity effects may also depend on factors such as the comparison group. Individuals could consider how their values compare to those in their local community, their city, their country, all of humanity, or to those of a range of outgroups, including immigrants, and religious, political, or age groups. Although value similarity effects have been shown to occur across ethnic and political boundaries (e.g., Hanel & Wolf, 2019; Wolf *et al*., 2019), the level of ingroup identification might moderate the impact of similarity information. For example, seeing a political outgroup emphasizes the same values in combatting COVID‐19 (e.g., ‘save lives’, ‘help each other’) may increase feelings of connectedness more strongly among people who identify less with their political ingroup, because their need for establishing ingroup distinctiveness is lower (e.g., Crisp & Beck, 2005). Hence, interventions aimed at mitigating the spread of COVID‐19 by highlighting value similarities may benefit from considering the role of ingroup identification.

## Conclusion

Existing evidence suggests that human values, and the extent to which they are shared by fellow citizens, are likely to be important factors for tackling the COVID‐19 crisis. Individuals who attach higher importance to self‐transcendence (e.g., responsibility) and conservation (e.g., security) values are likely to be more compliant with COVID‐19 behavioural guidelines. Moreover, perceiving that others share one's values is likely to elicit a sense of connectedness that may be crucial in promoting collective efforts to contain the pandemic.

Building on this evidence, communications to promote COVID‐19 mitigating behaviours could seek to tailor messages to the motives underlying people's values. For instance, highlighting how individuals can pursue openness values such as stimulation (e.g., online music concerts) or self‐enhancement values such as achievement (e.g., online courses) while adhering to COVID‐19 guidelines might be particularly effective among individuals attaching higher importance to such values. Similarly, interventions may also encourage individuals to reflect on links between their own values and COVID‐19 mitigating behaviours themselves, thereby embedding the behaviours more deeply in their values and facilitating long‐term commitment. Moreover, the media and policymakers could consider ways in which people's misperceptions of value differences with fellow citizens can be corrected. Reports of the public's compliance with guidelines may elicit higher feelings of social connectedness when they accurately emphasize the relatively high prevalence of compliers, rather than accentuating the minority of non‐compliers. Further, initiatives that promote (online) exchanges among individuals across society may also be beneficial for correcting such biases, and such work already exists (e.g., mycountrytalks.org). Overall, the abstract nature of values, and cross‐cultural agreement on their importance, suggests that they are well placed for developing and tailoring effective, global interventions to combat this pandemic.

## Conflict of interest

All authors declare no conflict of interest.

## Author contribution

Lukas Wolf (Conceptualization; Project administration; Writing – original draft; Writing – review & editing) Geoffrey Haddock (Supervision; Writing – review & editing) Antony S. R. Manstead (Supervision; Writing – review & editing) Gregory R. Maio (Conceptualization; Supervision; Writing – review & editing).
